# Regulation of astrocyte activity and immune response on graphene oxide-coated titanium by electrophoretic deposition

**DOI:** 10.3389/fbioe.2023.1261255

**Published:** 2023-10-03

**Authors:** Yong-Soo Han, Jun-Hwee Jang, Won-Seok Lee, Jun-Sung Oh, Eun-Jung Lee, Bo-Eun Yoon

**Affiliations:** ^1^ Department of Molecular Biology, College of Science and Technology, Dankook University, Cheonan, Republic of Korea; ^2^ Nano-Bio Medical Science, Graduate School, Dankook University, Cheonan, Republic of Korea; ^3^ Institute of Tissue Regeneration Engineering (ITREN), Dankook University, Cheonan, Republic of Korea; ^4^ Mechanobiology Dental Medicine Research Center, Dankook University, Cheonan, Republic of Korea

**Keywords:** astrocyte, anti-inflammatory cytokine, electrophoretic deposition, graphene oxide, titanium

## Abstract

**Introduction:** Astrocytes play crucial role in modulating immune response in the damaged central nervous system. Numerous studies have investigated the relationship between immune responses in astrocytes and brain diseases. However, the potential application of nanomaterials for alleviating neuroinflammation induced by astrocytes remains unexplored.

**Method:** In this study, we utilized electrophoretic deposition (EPD) to coat graphene oxide (GO) onto titanium (Ti) to enhance the bioactivity of Ti.

**Results:** We confirmed that GO-Ti could improve cell adhesion and proliferation of astrocytes with upregulated integrins and glial fibrillary acidic protein (GFAP) expression. Moreover, we observed that astrocytes on GO-Ti exhibited a heightened immune response when exposed to lipopolysaccharide (LPS). Although pro-inflammatory cytokines increased, anti-inflammatory cytokines and brain-derived neurotrophic factors involved in neuroprotective effects were also augmented through nuclear localization of the yes-associated protein (YAP) and nuclear factor kappa B (NF‐κB).

**Discussion:** Taken together, GO-Ti could enhance the neuroprotective function of astrocytes by upregulating the expression of anti-inflammatory cytokines and neuroprotective factors with improved cell adhesion and viability. Consequently, our findings suggest that GO-Ti has the potential to induce neuroprotective effects by regulating cell activity.

## Introduction

Astrocytes are the most abundant cells in the CNS and interact with neurons by releasing gliotransmitters such as ATP, glutamate, and γ-aminobutyric acid. These gliotransmitters could regulate neuronal activity and synaptic function (Perez-Alvarez and Araque, 2013; Chung et al., 2015; Phatnani and Maniatis, 2015; Lee et al., 2022). Moreover, astrocytes also release cytokines and participate in immune response in CNS, which is associated with neurodevelopmental and neurodegenerative diseases (Jensen et al., 2013; Colombo and Farina, 2016; Norden et al., 2016). Astrocyte activation was generally induced by pathological conditions and required for deriving inflammatory response by releasing cytokines. When astrocytes are activated, they could be changed in cell morphology and release pro-inflammatory cytokines such as interleukin (IL)-6, IL-1β and tumor necrosis factor (TNF)-α. However, these molecules could induce a chronic inflammatory response, which leads to neuronal damage. To alleviate this response, astrocytes could produce and release anti-inflammatory and neuroprotective molecules such as IL-10 and CXCL12, brain-derived neurotrophic factor (BDNF), and glial cell-derived neurotrophic factor (GDNF) (Farina et al., 2007; Farina, 2010; Czeh et al., 2011; Choi et al., 2014; Colombo and Farina, 2016). The balance of pro- and anti-inflammatory responses was crucial to maintain normal functions and prevent CNS pathological conditions. Therefore, we applied nanomaterials to modulate cellular activity related to the inflammatory response in astrocytes.

Titanium (Ti) has excellent mechanical properties such as a low elastic modulus, high corrosion resistance and high biocompatibility (Guo et al., 2006; Sidambe et al., 2012; Sidambe, 2014). Therefore, Ti is extensively used as a biomedical material for orthodontic mini-screws and bone joint implants. There are studies for surface modifications using physical or chemical treatment approaches to improve the bioactivity of Ti (Variola et al., 2009; Kate et al., 2019). Graphene has a two-dimensional planar honeycomb structure owing to the sp^2^ bonding of carbon atoms in a graphite layer. It exhibits excellent thermal conductivity, electrical conductivity, and electron mobility. Additionally, it has high tensile strength and good elasticity and does not lose its electrical properties even when bent or stretched (Geim and Novoselov, 2007; Adeel et al., 2018). Graphene becomes graphene oxide (GO) by oxidation. Its aqueous dispersibility, biocompatibility, and capacity to combine with biomolecules such as drugs and proteins can be substantially enhanced (Dreyer et al., 2010; Wu et al., 2015; Zhang et al., 2016). Graphene is exfoliated as a single layer with a significant number of oxygen functional groups after being oxidized by a strong acid such as sulfuric acid. Because of its 2D planar structure, single-layered graphene oxide has the advantage of improving the attachment dimension per unit volume for drug combinations and being uniform and thinly coated. Consequently, GO has primarily been used for coating or wrapping other compounds. In particular, the significant negative charge characteristic of GO in aqueous solutions adapts itself to electrophoretic deposition coating (EPD). The EPD is well-known for its stability and economical coating because it can produce a coating layer that is fast, uniform, and easy to control at room temperature. Moreover, EPD can be used for complex shapes from very small or large-area substrates and has a high potential for use in the surface modification of various implantable materials.

Nanomaterials and nanotechnology have been intensively explored to ameliorate neurodegenerative diseases by regulating inflammatory responses. Recent research in the CNS has demonstrated that nanomaterials can promote or inhibit the activity of nerve cells, stimulate the formation and growth of synapses, and repair synaptic connections (Place et al., 2009; Luo et al., 2011; Fabbro et al., 2013). Previous studies between neurons and graphene oxide reported that graphene oxide-titanium (GO-Ti) improves the viability of neurons and has less cytotoxicity than Ti (Fraczek-Szczypta et al., 2020). Graphene oxide induces morphological changes in astrocytes and regulates extracellular homeostasis via potassium channels (Chiacchiaretta et al., 2018). Moreover, releasing pro-inflammatory cytokines was reduced in a study for macrophages and graphene oxide, demonstrating anti-inflammatory effect (Hoyle et al., 2018).

In this study, we confirm morphological and functional changes such as inflammatory response in astrocytes on GO-Ti. Furthermore, we investigate nuclear transcription factors and mechano-transduction nuclear factor, a mechanism by which astrocytes convert into cellular activity in response to mechanical stimuli (Martino et al., 2018). Therefore, our study suggests that improving function with a nanomaterial could modulate astrocyte activity and be employed further in biomedical applications.

## Results

### Characterization of GO-Ti after EPD

**FIGURE 1:**
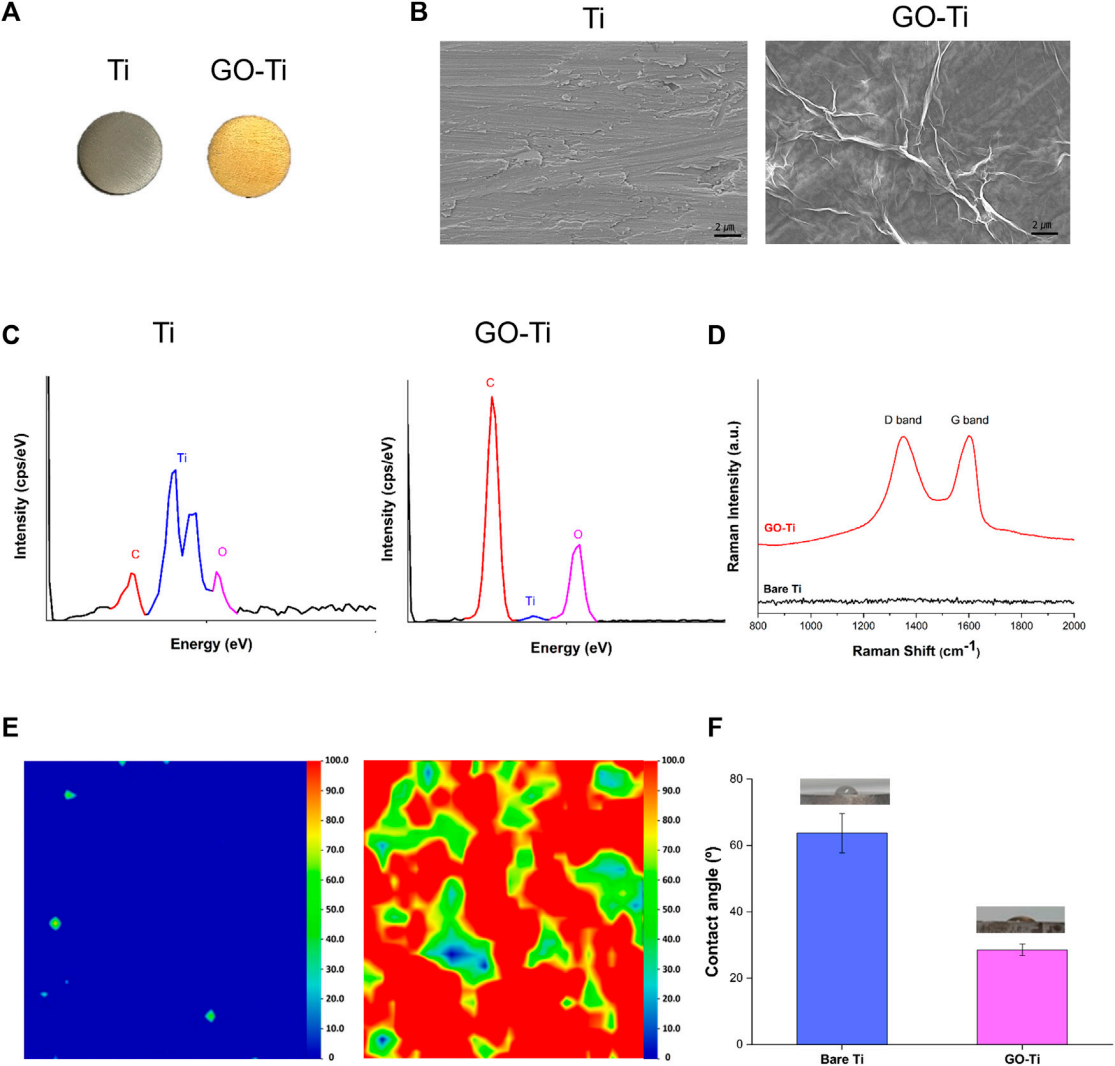
Characterization of Titanium (Ti) and Graphene Oxide coated Titanium (GO-Ti) **(A)** Optical images of Ti and GO-Ti; **(B)** The microstructure of Ti and GO-Ti obtained using field emission scanning electron microscopy (FE-SEM); **(C)** EDS spectrum of Ti after a treatment with GO; **(D)** Raman spectrum of Ti and GO-Ti; **(E)** Raman mapping of Ti and GO-Ti; **(F)** The contact angle of Ti and GO-Ti.

The differences in color between the two substrates are shown in Figure 1A. It was observed that GO was uniform and well-coated in yellow on bare Ti due to its brownish color. FE-SEM observations were conducted to analyze the surface modifications of the substrates (Figure 1B). Bare Ti showed a rough grinding surface, while GO-Ti substrate showed a deposited GO surface. The chemical components of the surface were investigated using EDS to confirm the GO coating on the Ti (Figure 1C). As Ti has a surface composed of TiO and TiC, Ti, O, and C were identified in bare Ti (Jung et al., 2016; Marcin Behunova et al., 2021). Like bare Ti, GO-Ti revealed three peaks indicating Ti, O, and C. It was confirmed that the GO coating relatively increased the intensity of the C and O elements compared to the intensity of the Ti peak.

The components of the graphene-coated surfaces were accurately analyzed using Raman spectroscopy (Claramunt et al., 2015; Perumbilavil et al., 2015). GO generally displays a D-band and G-band in the Raman spectrum. In Figure 1D, the D and G bands were not apparent in bare Ti but were observed in GO-Ti. In GO-Ti, the D band associated with the vibration of sp^3^ carbon atoms was observed in the range 1,340–1,350 cm^-1^, while a G band associated with the in-plane vibration of sp^2^ carbon atoms was detected at 1,600 cm^⁻1^ (Qin et al., 2014; Lopez-Diaz et al., 2017; Oh et al., 2021). The uniformity and quality of G” on the substrate surface were visualized by Raman mapping with an integrated intensity of the D and G bands (1,200–1700 cm^⁻1^) (Figure 1D). Because the Raman spectra were not detected in the D or G bands, the integrated intensity was between 0 and 100, determined based on the highest integrated intensity of the D and G bands among the several GO flask coatings. Raman mapping is depicted as blue to red images scaled from 0 to 100. The overall blue color mapping indicates that the total intensity is zero due to the absence of GO on bare Ti. The mapping of the GO-coated surface is mainly indicated by red and green colors. The color difference indicates the difference in the integrated intensity within the range, which is related to the thickness uniformity of the coated GO. Although the quality of the coated GO, such as thickness uniformity, was slightly different on the entire surface, the existence of GO on the Ti surface was clearly confirmed. Contact angle experiments were conducted to investigate the improvement in the hydrophilicity of the surface after GO deposition (Zuo et al., 2013; Oh et al., 2021). In Figure 1F, the contact angle of Ti was 63.74° ⁻5.92°, and that of GO-Ti was 28.52° ± 1.74°. This indicates that when the GO was deposited, the surface of Ti became more hydrophilic. These findings suggest that the GO-coated layer has higher hydrophilicity and better biocompatibility to brain cells than bare Ti.

### Increased cell area and cell adhesion of astrocytes on GO-Ti

**FIGURE 2:**
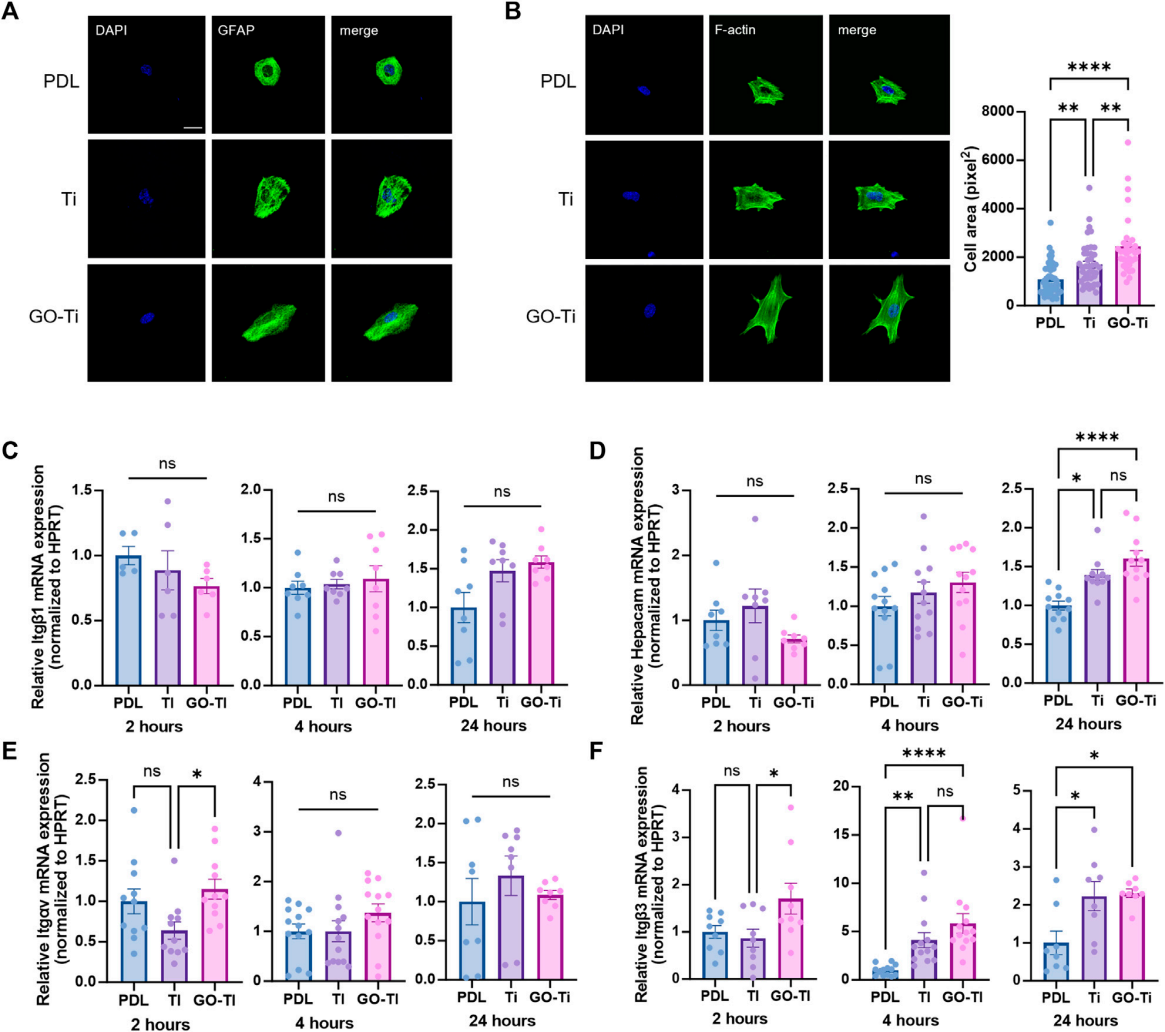
Cell morphology and attachment of astrocytes on GO-Ti. **(A)** Representative images of immunocytochemistry with GFAP (green) in primary astrocytes. **(B)** Representative images of immunocytochemistry with phalloidin (green) in primary astrocytes (left) and cell area of primary astrocytes by image analysis with region of interest (right). **(C–F)** mRNA expression of adhesion molecules in primary astrocytes at 2, 4 and 24 h by qRT-PCR. Expression of target mRNA was normalized by *Hprt.* **(C)** mRNA expression of *Itgb1* in primary astrocytes. **(D)** mRNA expression of *Hepacam* in primary astrocytes. **(E)** mRNA expression of *Itgav* in primary astrocytes. **(F)** mRNA expression of *Itgb3* in primary astrocytes. *p < 0.05, **p < 0.01, ***p < 0.001, ****p < 0.0001. n = 4 from four independent cell preparations and experiments.

To investigate astrocyte cell area and adhesion on Poly-D lysine (PDL), Ti and GO-Ti, we stained glial fibrillary acidic protein (GFAP), an astrocyte marker, and F-actin with F-actin using Phalloidin. First, we verified that most primary culture cells were astrocytes (Supplementary Figure S1). Next, we confirmed that astrocytes were attached on PDL, Ti, and GO-Ti (Figure 2A). Also, astrocytes on GO-Ti had a wider cell area than PDL and Ti (Figure 2B). A prior study revealed that increased cell area enhanced cell adhesion and proliferation (Ruiz et al., 2011; Pan et al., 2016). Therefore, we confirmed that the mRNA expression levels of integrin (*Itg*) and glial cell adhesion molecule (*G-CAM; Hepacam*) (Figures 2C–F). There was no difference in *Itgb1* mRNA expression levels after 2, 4, or 24 h seeding (Figure 2C). G-CAM mRNA expression was increased on GO-Ti than PDL after 24 h (Figure 2D). The *Itgav* mRNA expression was shown to be greater on GO-Ti than Ti after 2 h (Figure 2E). The *Itgb3* mRNA expression was elevated not only on GO-Ti at 2 h, but also on PDL at 4 h and 24 h (Figure 2F). Therefore, we suggest that astrocytes have a faster and stronger cell adhesion on GO-Ti than PDL or Ti and that the increased astrocyte cell area could influence cell growth and proliferation.

### Enhanced viability, proliferation and activity of astrocytes on GO-Ti

**FIGURE 3:**
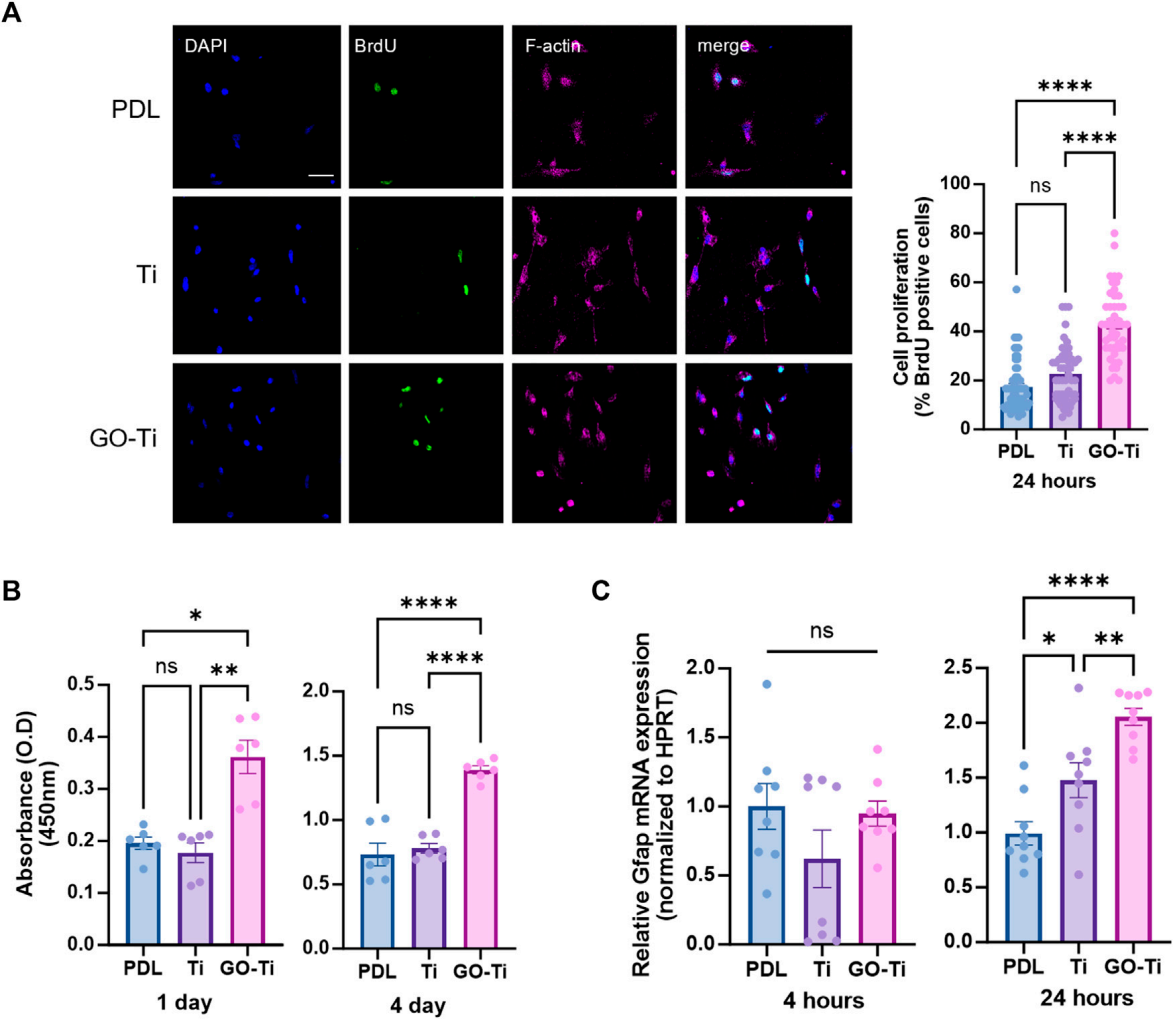
Cell survival and activity of astrocytes on GO-Ti. **(A)** Representative images of immunocytochemistry with BrdU (green) and phalloidin (magenta) in primary astrocytes (left) and percentage of primary astrocytes positive for BrdU (right). **(B)** Cell viability was assayed with CCK-8 for days 1 and 4. **(C)** mRNA expression of *Gfap* in primary astrocytes by qRT-PCR at 4 and 24 h mRNA expression of GFAP was normalized by *Hprt*. *p < 0.05, **p < 0.01, ***p < 0.001, ****p < 0.0001. N = 3 from three independent cell preparations and experiments.

Cell growth due to increased cellular area was associated with cell proliferation on GO-Ti (Ruiz et al., 2011). To determine whether cell proliferation was affected by GO-Ti, we stained astrocytes with cell proliferation marker, Bromodeoxyuridine (BrdU) (Figure 3A). The number of astrocytes stained with BrdU on GO-Ti was higher than PDL and Ti. Following that, we investigated cell viability using CCK-8 to determine whether GO-Ti could affect astrocyte proliferation (Figure 3B). On days 1 and 4, GO-Ti had better cell viability than PDL and Ti. Furthermore, we confirmed the mRNA expression level of GFAP, an astrocyte marker (Figure 3C). Although there was no difference at 4 h, we observed that the mRNA expression level of GFAP in astrocytes on GO-Ti increased at 24 h. Taken together, we suggest that GO-Ti can upregulate *Gfap* mRNA expression and improve cell proliferation in astrocytes.

### Efficient immune response of astrocytes on GO-Ti

**FIGURE 4:**
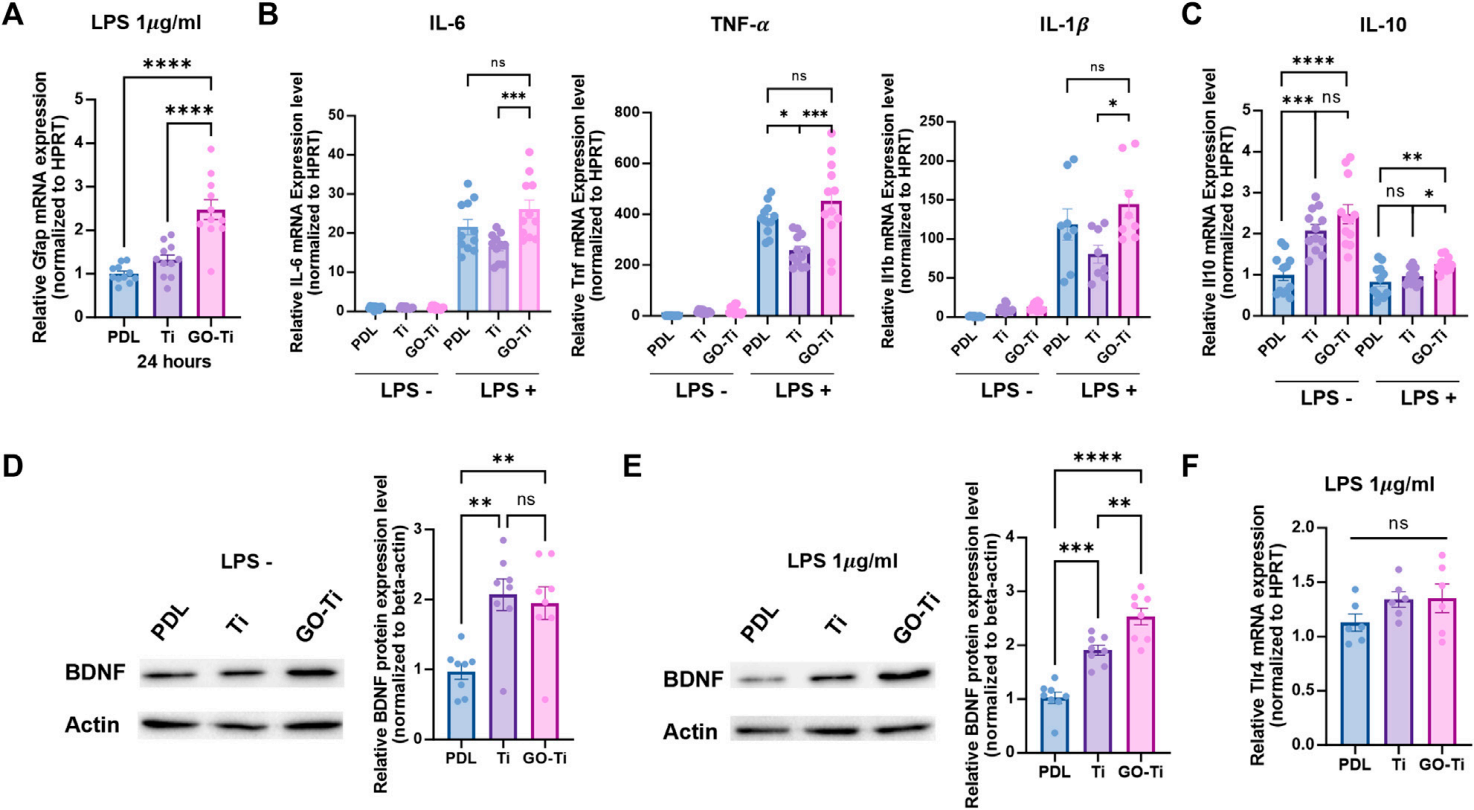
The immune response of astrocytes on GO-Ti. **(A)** mRNA expression of Gfap in primary astrocytes by qRT-PCR after treatment with LPS. **(B)** mRNA expression of pro-inflammatory cytokines such as *Il6*, *Tnf* and *Il1b* in primary astrocytes by qRT-PCR without or with LPS. **(C)** mRNA expression of anti-inflammatory cytokines such as *Il10* in primary astrocytes by qRT-PCR without or with LPS. **(D)** Protein expression of BDNF (37 kDa) in primary astrocytes without LPS. **(E)** Protein expression of BDNF (37 kDa) in primary astrocytes with LPS. Expression of BDNF protein was normalized by β -actin (48 kDa). **(F)** mRNA expression of *Tlr4* in primary astrocytes by qRT-PCR with LPS. Expression of target mRNA was normalized by *Hprt*. *p < 0.05, **p < 0.01, ***p < 0.001, ****p < 0.0001. n = 3 from three independent cell preparations.

In many pathological conditions in CNS such as Alzheimer’s and Parkinson’s disease, astrocytes could be induced to trigger primarily immune response and neurotoxicity (Choi et al., 2014; Phatnani and Maniatis, 2015; Colombo and Farina, 2016). Astrocytes produce proinflammatory cytokines to trigger an inflammatory response and eliminate pathogens or damaged cells. Astrocytes release anti-inflammatory cytokines to suppress chronic inflammatory responses and maintain immune system equilibrium (Jensen et al., 2013; Norden et al., 2016). Here, we incubated astrocytes with lipopolysaccharide (LPS) (1 μg/mL) for 4 h to induce an immune response. First, we determined whether the astrocyte cultured on GO-Ti could induce immune response by LPS treatment. GFAP mRNA expression was higher on GO-Ti than on PDL and Ti (Figure 4A). We also investigated the inflammatory response in astrocytes activated by LPS to GO-Ti changes (Figures 4B,C). When LPS was not treated, there was no variation in the mRNA expression levels of pro-inflammatory cytokines such as *Il6, Tnf,* and *Il1b*. In contrast, the mRNA expression levels of the three pro-inflammatory cytokines on GO-Ti increased following LPS treatment compared to Ti. However, there was no difference between PDL and GO-Ti (Figure 4B). These results showed that Ti decreased the basal immune response of astrocytes, whereas GO-Ti exhibited a basal immune reaction in astrocytes. The mRNA expression level of *Il10*, an anti-inflammatory cytokine, was enhanced in astrocytes on GO-Ti compared to PDL in both circumstances, with and without LPS. When LPS was treated to Ti and GO-Ti, the mRNA expression level of Il10 increased. Furthermore, we investigated the levels of protein expression of BDNF, a neurotrophic factor secreted by astrocytes, on GO-Ti (Figures 4D,E). When LPS was not treated, BDNF protein expression was increased on Ti and GO-Ti compared to PDL, but there was no difference between Ti and GO-Ti (Figure 4D). When treated with LPS, GO-Ti enhanced BDNF protein expression in astrocytes compared to PDL and Ti (Figure 4E). To establish if the three groups had distinct immunological responses due to improved LPS sensitivity, the mRNA expression level of Toll-like receptor (*Tlr*) 4, a receptor to which LPS binds, was studied (Figure 4F). There was no difference in the PDL, Ti, or GO-Ti after LPS treatment. Therefore, we suggest astrocytes on GO-Ti could have an efficient and balanced inflammatory response.

### YAP and NF- κ B of astrocytes activated on GO-Ti

**FIGURE 5:**
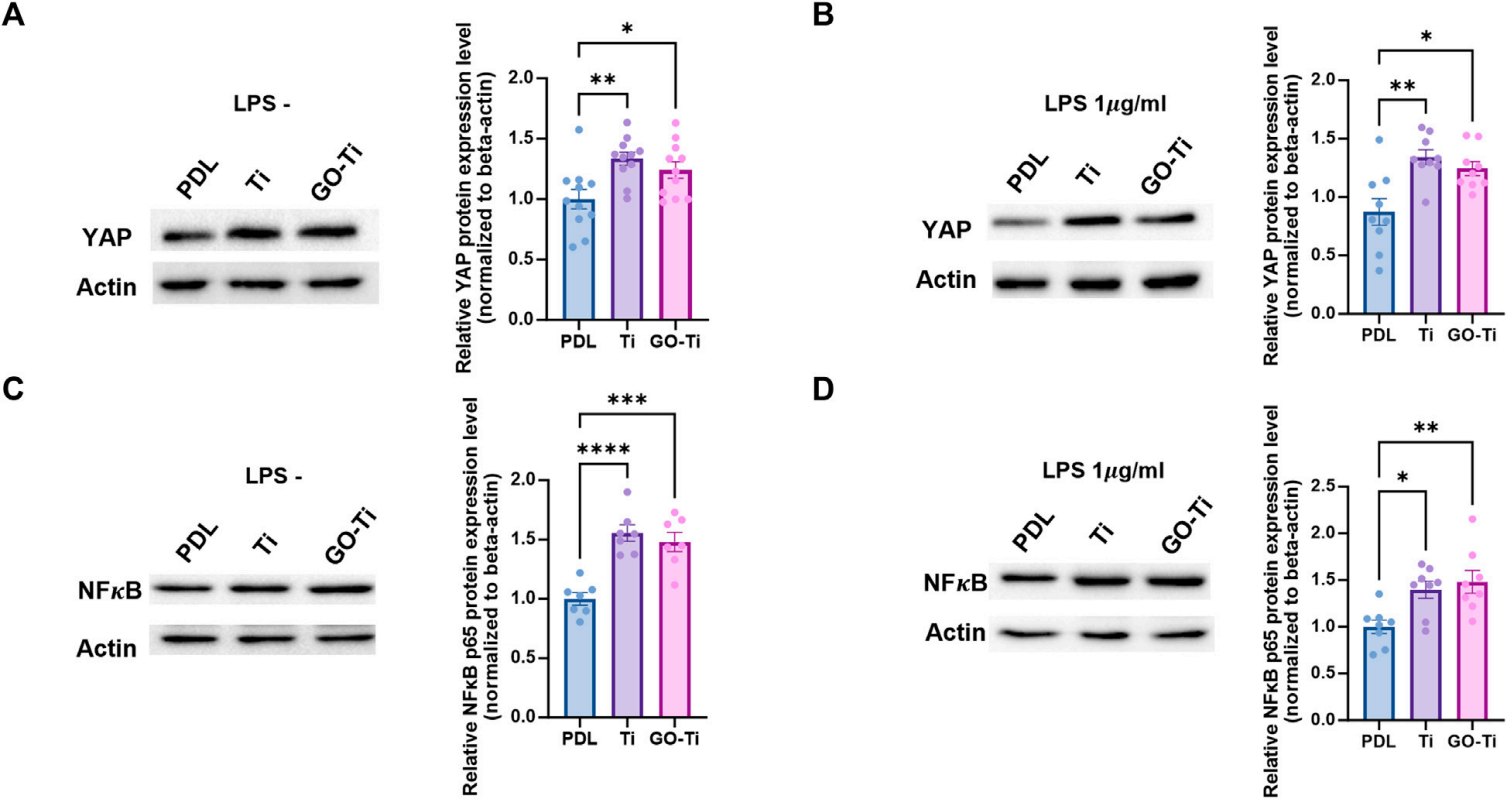
Total protein expression levels of YAP and NF𝜅B in astrocytes on GO-Ti. **(A)** Representative images of western blot (YAP) 202 in primary astrocytes without LPS (left) and protein expression of YAP (65kDa) in primary astrocytes without LPS (right). **(B)** 203 Representative images of western blot (YAP) in primary astrocytes with LPS (left) and protein expression of YAP (65 kDa) in primary 204 astrocytes with LPS (right). **(C)** Representative images of western blot (NF𝜅B) in primary astrocytes without LPS (left) and protein 205 expression of NF𝜅B (65 kDa) in primary astrocytes without LPS (right). **(D)** Representative images of western blot (NF𝜅B) in primary 206 astrocytes with LPS (left) and protein expression of NF𝜅B (65 kDa) in primary astrocytes with LPS (right). Expression of YAP and NF𝜅B 207 protein was normalized by *β*-actin (48 kDa). *p < 0.05, **p < 0.01, ***p < 0.001, ****p < 0.0001. n = 3 from three independent cell 208 preparations.

**FIGURE 6:**
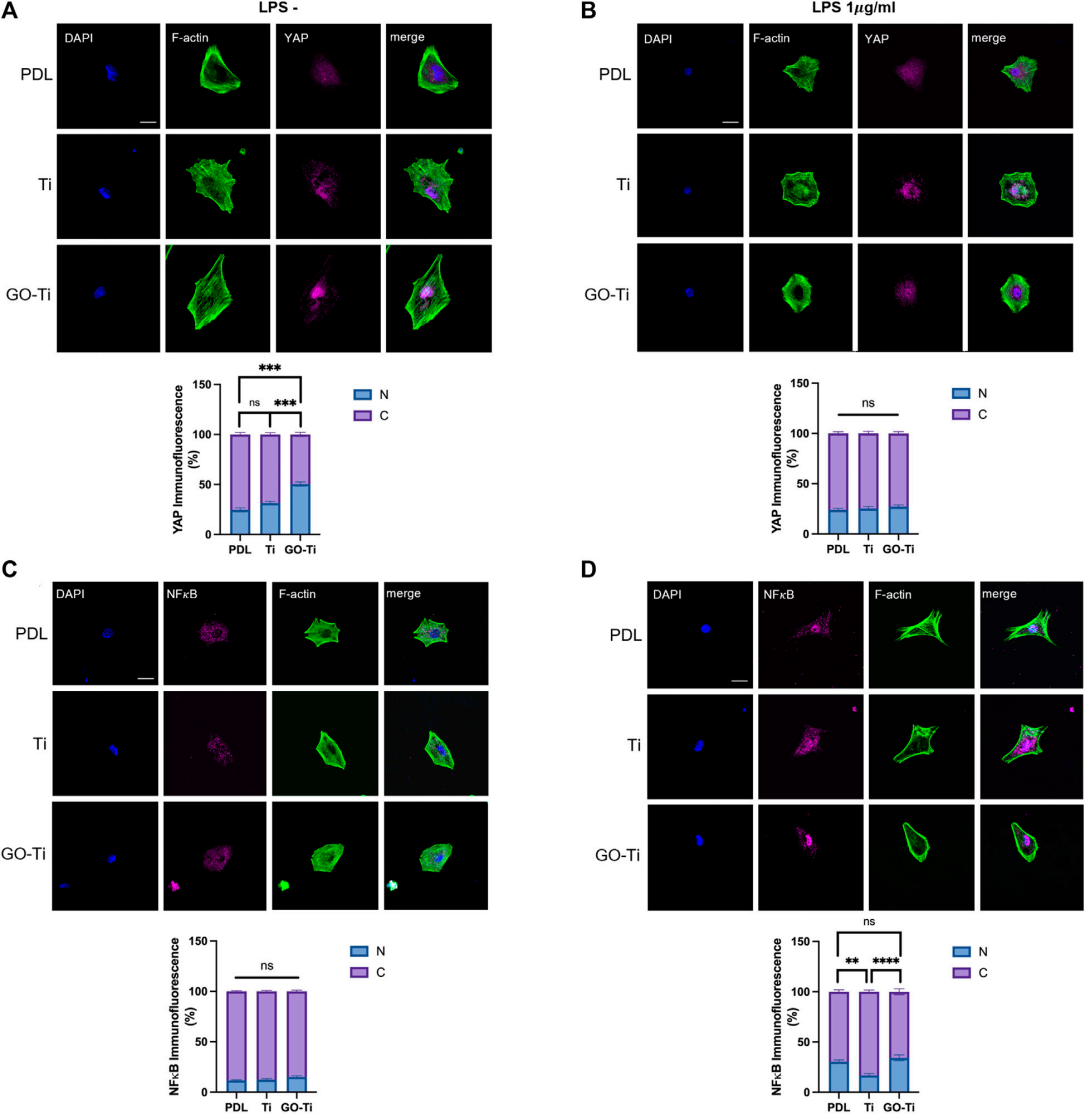
Ratio of YAP and NF𝜅B located in the nucleus of astrocytes on GO-Ti. **(A,B)** Representative images of 242 immunocytochemistry with phalloidin (green) and YAP (magenta) in primary astrocytes without LPS (left) or with LPS (right) and 243 percentage of YAP located in the nucleus compared to YAP located in the cytosol without LPS (left) or with (right) LPS by image 244 analysis. **(C,D)** Representative images of immunocytochemistry with phalloidin (green) and NF𝜅B (magenta) in primary astrocytes 245 without LPS (left) or with LPS (right) and percentage of NF𝜅B located in the nucleus compared to NF𝜅B located in the cytosol without 246 LPS (left) or with LPS (right) by image analysis. *p <0.05, **p <0.01, ***p <0.001, ****p <0.0001. For LPS (-) YAP group, n means 247 individual cell numbers. PDL; n = 35, Ti; n = 44 and GO-Ti; n = 48. For LPS (+) YAP group, PDL; n = 35, Ti; n = 42 and GO-Ti; n = 34. For 248 LPS (-) NF𝜅B group, PDL; n = 42, Ti; n = 44 and GO-Ti; n = 44. For LPS (+) NF𝜅B group, PDL; n = 50, Ti; n = 36 and GO-Ti; n = 40.

When astrocytes on PDL, Ti, and GO-Ti were incubated with LPS, the mRNA expression of Tlr4 exhibited no difference (Figure 4F). Therefore, we investigated other factors for this response. We confirmed mechanotransduction, a process by which mechanical stimulation of GO-Ti influences cell chemical activity as well as nuclear transcription factors. First, we studied representative mechanotransduction factors, yes-associated protein 1 (YAP), which is a major downstream effector of the Hippo signaling pathway. YAP regulates cell proliferation and remodeling of injured tissues as well as cell growth, survival, differentiation, morphological changes, and stem cell renewal (Jin et al., 2020). YAP can also be regulated by the extracellular matrix (Dupont et al., 2011). The nuclear transcription factor NF-κB is widely known to be a signaling pathway that induces an immune response. NF-κB induces immune responses by regulating the expression of cytokines, chemokines, and adhesion molecules. Previous studies have revealed that NF-κB induces both pro-inflammation and anti-inflammation (Lawrence, 2009). We confirmed the protein expression levels of YAP and NF-κB on PDL, Ti, and GO-Ti (Figures 5A–D). Both with and without LPS, the protein expression level of YAP was increased on Ti and GO-Ti compared to PDL. However, there was no difference in the protein expression levels of YAP between Ti and GO-Ti (Figures 5A,B). Likewise, the protein expression level of NF-κB was observed in the same pattern as YAP (Figures 5C, D). It is known that the nuclear transcription factors YAP and NF-κB located in the cytoplasm became active when translocated into the nucleus (Lawrence, 2009; Dupont et al., 2011; Jin et al., 2020). Here, we confirmed the ratio of YAP and NF-κB proteins located in the cytoplasm and nucleus (Figures 6A–D). When LPS was not treated, the ratio of YAP protein located in the nucleus increased on GO-Ti rather than PDL and Ti. When LPS was not treated, the ratio of YAP proteins located in the nucleus increased on GO-Ti rather than PDL and Ti (Figure 6A). However, after LPS treatment, the ratio of YAP proteins located in both the cytoplasm and nucleus showed no differences between PDL, Ti, and GO-Ti (Figure 6B). The ratio of NF-κB proteins located in both the cytoplasm and nucleus was not different on PDL, Ti, and GO-Ti when not treated with LPS (Figure 6C). However, after LPS treatment, the ratio of NF-κB proteins located in the nucleus was higher on PDL and GO-Ti than Ti (Figure 6D). Therefore, our findings imply that enhanced YAP activity in astrocytes stimulates adhesion, growth, and proliferation. Moreover, when astrocytes on GO-Ti induced an immune response, the increased activity of NF-κB protein can induce pro- and anti-inflammatory responses.

## Discussion

GO is known to have a positive effect on cell adhesion and proliferation (Ghorbani et al., 2019); herein, we could expect the enhanced adhesion behavior of astrocytes because GO was well coated on the Ti substrate, which was demonstrated through FE- SEM and Raman analysis (Figures 1B–E). In addition, many studies have reported that the surface provides more favorable conditions for cell adhesion and protein adhesion when the angle of water droplets is less than 3°–40° (Desai and Hubbell, 1991; Han et al., 1996). Although bare Ti is biocompatible, when coated with GO, the water droplet contact angle is expected to decrease considerably (Figure 1F), resulting in hydrophilicity to cause better cell and protein adherence.

The characteristics of GO are beneficial as a biomaterial and have been actively studied for bioengineering. EPD is also one of the preferred coating methods, owing to its simplicity and speed. Prior to this study, we performed a biological evaluation of GO-coated Ti using EPD for complex tissue engineering using human mesenchymal stem cells. Our previous study confirmed the positive effect of GO-modified surfaces on controlled drug loading, releasing capacity and osteogenic differentiation (Oh et al., 2021). Several studies have shown that electrical signal stimulation by GO can affect the proliferation of nerve cells that send and receive signals (Parandeh et al., 2020; Wang et al., 2020; Yao et al., 2021). However, it is also known that GO does not always have a beneficial effect on astrocytes. When astrocytes were incubated with GO, the substrate could increase plasma membrane cholesterol and disrupt Ca^2+^ homeostasis (Bramini et al., 2019). Therefore, we used a method of coating titanium through EPD to make GO noninvasively. Also, the GO-coated biometal is anticipated to have the potential to be used as a component of implanted devices such as neural probes and needles. However, research on the interaction of these GO-coated materials and astrocytes is sparse when compared to findings on the effects of bone formation and wound healing on nerve cell differentiation and regeneration. Therefore, we attempted to validate its potential as an implantable material for neurological therapy in this study by assessing the effect of GO-modified metal implants on astrocyte behaviors such as proliferation, gene expression, immunological responses, and so on.

In this study, we investigated the interaction between astrocytes and GO-Ti. We confirmed that the upregulated expression of integrin leads to an increase in the astrocytic area and improved proliferation on GO-Ti. These cell area expansions and the increase in integrin are closely associated with focal adhesion (Cavalcanti-Adam et al., 2007; Delon et al., 2009). Furthermore, we propose that these results are induced by upregulated focal adhesion through nuclear localization of YAP, which is crucial for extracellular sensing and mechanotransduction (Dupont et al., 2011; Jin et al., 2020). Therefore, we suggest GO-Ti could induce cell proliferation via increased focal adhesion and YAP of nuclear localization. Additionally, we observed an increased expression of the GFAP gene in astrocytes on GO-Ti, indicating changes in cellular activity. Astrocytes activity with increased GFAP expression was closely associated with inflammatory response by releasing cytokines and neurotrophic effects via neurotrophic factor (Giovannoni and Quintana, 2020). Therefore, we exposed LPS to astrocytes cultured on GO-Ti to induce cellular activity and observed the expression of inflammatory cytokines and neurotrophic factors. Although we observed an increase pro-inflammatory cytokines gene expression level, we confirmed an increase in the gene expression of anti-inflammatory cytokines. Also, we confirmed that the BDNF increased in astrocytes. It is well known that maintaining a balance between pro-inflammatory and anti-inflammatory responses plays a crucial role in sustaining health and homeostasis. However, continuous pro-inflammatory response could induce in chronic inflammation by releasing pro-inflammatory cytokines (Cicchese et al., 2018). Moreover, uncontrolled acute inflammation could be chronic inflammation. For this reason, inducing anti-inflammatory responses was vital for the prevention of chronic inflammation (Chen et al., 2018). Therefore, we indicate that GO-Ti could regulate balance of pro- and anti-inflammatory response and induce neurotrophic effects on astrocytes. Taken together, we propose that GO-Ti could be potential nanomaterials for sustaining balance between pro- and anti-inflammatory responses of astrocytes.

## Methods and materials

### Electrophoretic deposition (EPD) with graphene oxide

Grade 2 titanium (Titanart, Korea) was selected as the titanium plate. Prior to the experiment, it was polished with 800, 1,200, and 2000 grit silicon carbide paper (Daesung, Korea) to obtain an even surface of titanium. The residue was removed from acetone, ethanol (Duksan, Korea), and DW by ultrasonic washing. A single layer of graphene oxide (GO; Graphene Supermarket, USA) was used, and high-purity ethanol was used as the electrolyte. GO (500 ug/mL was prepared using 80% EtOH, and ultrasonic wave treatment was performed for 15 min to obtain a uniform electrolyte. The Ti substrate was placed in an electrolyte maintained at 1 cm from the anode. A Ti substrate was also used as the anode. For the EPD coating, an anode EPD process, in which electricity passes at a current of 10 mA for 1 min, was performed using a DC power source (TPM series, TOYOTECH, Korea) because GO has a negative charge. Subsequently, it was ultrasonically washed for 5 min in the order of acetone, ethanol, and water, and then dried. For the *in vitro* tests, the coated material was sterilized with a 70% ethanol solution.

### Characterization

Field-emission scanning electron microscopy (FE-SEM, Zeiss 300 Gemini, Germany) and energy-dispersive spectroscopy (EDS, UltraDry, Thermo USA) were used to confirm the coating surface and the elements of bare Ti and GO-Ti. After attaching bare Ti and GO-Ti to the stage, the surface was observed at 5,000 × magnification at 5 kV. The EDS element analysis was also fixed at 5,000 magnifications at 5 kV, and the components and intensities of Ti, C, and O were analyzed in bare Ti and GO-Ti by line scan. Raman spectroscopy was used to determine the presence of graphene. Excitation was performed at 532 nm to evaluate the bare Ti and GO-Ti, and the measurement range was set from 800 cm^-1^ to 2000 cm^-1^. In addition, the GO coating on the surface was imaged using Raman intensity through Raman mapping. For mapping, the laser power was set to 2 mW, the width was 50 μm × 50 μm, and the Raman shift was measured in the range of 1,200–1700 (cm^⁻1^). The hydrophilicity of the bare Ti and GO-Ti surfaces was evaluated by measuring the contact angle. The hydrophilicity of the surface was compared by dropping water droplets onto the surface of each material to measure the inner angle of the water droplets.

### Animals

C57BL/6 mice were used to culture primary astrocytes. All experiments in this study were conducted in accordance with the guidelines for animal accordance at Dankook University (Animal Experiment Approval No. Dankook University 19-016, Cheonan, Korea).

### Primary astrocyte culture

To culture primary astrocytes, the brains of C57BL/6 mice were isolated at postnatal days 0–2. To obtain the cerebral cortex for use in the experiment, the cerebrum was separated, and the medulla was removed. The cerebral cortex was then pulverized by pipetting and cultured in a culture dish coated with 0.1 mg/mL Poly D-lysine (PDL, #354210, Corning). The culture medium for obtaining astrocytes was high-glucose Dulbecco’s modified Eagle medium (#LM001, Welgene) with 10% fetal bovine serum (#S001-07, Welgene), 10% horse serum (#26050-088, Gibco), and 1% penicillin/streptomycin (#LS202-02, Welgene) 05, Welgene). Primary astrocytes were incubated at 37°C and 5% CO_2_ for 3 days.

### Astrocytes culture on GO-Ti

Primary astrocytes cultured in a culture dish were reacted with 1x trypsin-EDTA solution (Welgene) at 37°C for 3 min and suspended by pipetting. The suspended astrocytes were then placed in a 15 mL conical tube (SPL) and centrifuged at 2,000 rpm at 4°C for 10 min. The supernatant was removed, and 1 mL of astrocyte culture medium was added, followed by pipetting of single cells. Astrocytes were dispensed with a culture solution on 0.1 mg/mL poly D-lysine-coated cover glass, Ti, and GO-Ti. The astrocytes were then incubated at 37°C and 5% CO_2_ for 24 h.

### Immunocytochemistry

After removing the culture medium of astrocytes cultured on PDL-cover slips, Ti, and GO-Ti in a 24-well plate (SPL), they were washed twice with 1 × PBS for 5 min. Subsequently, astrocytes were fixed by treatment with 4% paraformaldehyde (PFA) for 30 min. The fixed astrocytes were washed three times for 5 min with 1x PBS, and PBS (blocking solution) containing 2% normal goat serum (#005-00-121, Jackson Immunoresearch) and 0.3% Triton X-100 were added at room temperature for 1 h. Blocking was performed using a shaker for 30 min. After blocking, the primary antibody was diluted in the blocking solution and incubated on a shaker at 4°C for 20 h. The primary antibodies used were as follows: rabbit monoclonal anti-yes-associated protein 1 (YAP, #14074, Cell Signaling), mouse monoclonal anti-NF- κ B p65 protein (#6956, Cell Signaling), mouse monoclonal anti-5′-Bromo-2-deoxyuridine (BrdU, #B2531, Sigma-Aldrich), chicken polyclonal anti-GFAP (#AB5541, Millipore Bioscience Research). F-actin was stained using Alexa Fluor™ 488-conjugated phalloidin (#A12379, Invitrogen™), and F-actin was stained using Alexa Fluor™ 594-conjugated phalloidin (#A12381, Invitrogen™). Then, the primary antibody solution was removed, and the cells were washed thrice for 5 min with 1x PBS. The secondary antibody was diluted in blocking solution, reacted at room temperature for 1 h, and washed thrice for 5 min with 1 × PBS. The secondary antibodies used were as follows: Alexa Fluor 594-conjugated goat polyclonal anti-rabbit (#111-585-003, Jackson ImmunoResearch Inc.), Alexa Fluor 488-conjugated goat polyclonal anti-chicken (#103-545-155, Jackson ImmunoResearch Inc.), and Alexa Fluor™ 488-conjugated goat polyclonal anti-mouse (#115-545-003, Jackson ImmunoResearch Inc.). DAPI (6-diamidino-2-phenylindole) was diluted in 1x PBS and incubated at room temperature for 5 min. After the reaction was completed, the cells were washed for 5 min with 1x PBS. Astrocytes on the PDL-cover slip, Ti, and GO-Ti were fixed using Dako Faramount Mounting Medium (#S3023, Dako) for microscopic observation. Images were acquired using a confocal microscope (Zeiss LSM700) and ImageJ software was used for image analysis. The fluorescence intensity of YAP and NFκB was analyzed as follows: YAP and NF κ B fluorescence intensity (a.u.) = cell intensity - (area × mean background fluorescence), and the nucleus/cytoplasm ratio was analyzed the nucleus positive protein intensity/total protein intensity ratio.

### Measurement of cell viability and proliferation

The Cell Counting Kit-8 (CCK-8, #ALX-850-039-KI01, ENZO) was used to measure cell viability and proliferation. The culture medium of astrocytes (2 × 10^5^cells/well) cultured in 0.1 mg/mL PDL-cover slip, Ti and GO-Ti in a 24-well plate (SPL) was removed. The cells were then washed twice with 1 × PBS. In each well, 450 μL of astrocyte culture medium and 50 μL of CCK-8 solution was added and reacted at 37°C and 5% CO_2_ for 3 h. The absorbance was measured at 450 nm using a microplate reader (BIOTEK). The values calculated for cell viability and proliferation were as follows: O.D value of the sample–O.D value of the blank (culture medium + CCK-8 solution).

### Analysis of mRNA expression by qRT-PCR

#### RNA extraction

After removing the culture solution of astrocytes cultured in PDL-cover slips, Ti, and GO-Ti in a 6-well plate (SPL), they were washed twice with 1 × PBS. Then, 1 mL of Tri-RNA reagent (#FATRR 001, Favorgen) was added and reacted at room temperature for 5 min. Primary astrocytes were suspended by pipetting, and 200 μL of chloroform was added for protein denaturation, followed by vortexing and reaction at room temperature for 3 min. Upon completion of the reaction, centrifugation was performed at 13,000 rpm and 4°C for 15 min. Then, 200 μL of the supernatant was transferred to a new 1.5 mL tube, 200 μL of isopropanol was added, vortexed, and incubated at −20°C for 30 min. Upon completion of the reaction, centrifugation was performed at 13,000 rpm for 20 min at 4°C and the supernatant was removed. Next, 400 μL of 70% ethanol was added, and centrifugation was performed twice at 13,000 rpm at 4°C for 7 min. After removing the supernatant, 12 μL of DEPC-treated water (#AM9915G, Invitrogen) was added and dissolved. RNA purity and concentration were measured using a NanoDrop ND-1000 spectrophotometer (Thermo Scientific).

#### cDNA synthesis

To match the RNA concentration of each sample, the total RNA concentration was diluted with DPEC-treated water to 1 μg/10 μL. Next, 1 μL of Oligo dT primer (500 μg/mL) and 1 μL of 10 mM dNTP mix (10 mM each of dATP, dGTP, dCTP, and dTTP at neutral pH) were added and reacted at 65°C for 5 min. After the reaction was complete, the solution was stabilized on ice for 3 min. Then, 4 μL of 5 × First-Strand Buffer and 2 μL of 0.1 M DTT were added and reacted at 37°C for 2 min. After completion of the reaction, 1 μL of 200 units M-MLV (#28025013, Invitrogen™) was added, and reverse transcription was performed at 37°C for 50 min and at 70°C for 15 min.

#### qRT-PCR, quantitative real-time PCR

To compare the mRNA expression levels of genes, the CFX connect Real-time PCR Detection System (Bio-Rad) was used. For qRT-PCR of the sample, SYBR Green Realtime PCR Master Mix (#QPK-201, Toyobo) 10 μL, forward primer (10 μM) 0.6 μL and reverse primer (10 μM) 0.6 μL, cDNA 2 μL was added. To adjust the total volume to 20 μL, 6.8 μL of DEPC-treated water was added. Hypoxanthine-guanine phosphoribosyl transferase (Hprt), a housekeeping gene, was used to quantify the relative expression of the target gene. For PCR, pre-denaturation was performed at 95°C for 30 s followed by denaturation at 95°C for 5 s. Next, the primer and cDNA were reacted for 10 s at an annealing temperature suitable for the primer, followed by extension at 72°C for 15 s. The PCR cycle was repeated 40 times. The sequences of primers used to check mRNA expression levels by qRT-PCR are listed in a table (Table 1). Relative target gene mRNA expression levels were normalized using *Hprt*, and the data were analyzed using Microsoft Excel and GraphPad Prism 9.

**TABLE 1:** Primer sequences in qRT-PCR.

Primer	Sequence (5′-3′)
*Itgb1* forward	GCA​ACG​CAT​ATC​TGG​AAA​CTT​G
*Itgb1* reverse	CAA​AGT​GAA​ACC​CAG​CAT​CC
*Hepacam* forward	TCC​TTG​CTT​CTC​AGC​GAC​CT
*Hepacam*reverse	TAC​CTG​CGG​CCT​TGA​AAT​GG
*Itgav* forward	CAA​TTA​GCA​ACA​CGG​ACT​GC
*Itgav* reverse	CGT​CAC​CAT​TGA​AGT​CTC​CC
*Itgb3* forward	TTT​GAG​GAA​GAA​CGA​GCC​AG
*Itgb3* reverse	CCC​GGT​AGG​TGA​TAT​TGG​TG
*Gfap* forward	GAA​GCT​CCA​AGA​TGA​AAC​CAA​C
*Gfap* reverse	TCC​AGC​GAT​TCA​ACC​TTT​CTC
*Il6* forward	GTT​CTC​TGG​GAA​ATC​GTG​GA
*Il6* reverse	CTC​GAA​GGA​CTC​TGG​CTT​TG
*Tnf* forward	ATG​GCC​TCC​CTC​TCA​TCA​GT
*Tnf* reverse	CTC​CTC​CAC​TTG​GTG​GTT​TG
*Il1b* forward	CAG​CTC​ATA​TGG​GTC​CGA​CA
*Il1b* reverse	CTG​TGT​CTT​TCC​CGT​GGA​CC
*Il10* forward	CCC​TTT​GCT​ATG​GTG​TCC​TT
*Il10* reverse	TGG​TTT​CTC​TTC​CCA​AGA​CC
*Tlr4* forward	CAACTCATCCAGGAAGGC
*Tlr4* reverse	*GAA​GGC​GAT​ACA​ATT​CCA​CC*
*Hprt* forward	GCT​GGT​GAA​AAG​GAC​CTC​T
*Hprt* reverse	CAC​AGG​ACT​AGA​ACA​CCT​GC

### Protein expression analysis by western blot

#### Protein extraction

After removing the culture medium of astrocytes cultured in PDL-cover slips, Ti, and GO-Ti in a 6-well plate (SPL), the cells were washed twice with PBS. Then, 500 μL of radioimmunoprecipitation (RIPA) assay buffer was dispensed and the cells were scraped with a scraper. This was transferred to a 1.5 mL tube and centrifuged at 13,000 rpm at 4°C for 20 min. The supernatant containing the target protein was obtained and the protein was quantified using the PierceTM BCA Protein Assay Kit (#23225, Thermo Scientific).

### Western blot

The extracted protein was adjusted to 15 μg for each sample and reacted with 5× sample buffer (60 mM Tris-HCl, 2% SDS, 25% glycerol, 5% β-mercaptoethanol, and 0.1% bromophenol blue) at 100°C. for 5 min. Electrophoresis was performed using 12% sodium dodecyl sulfate-polyacrylamide gel electrophoresis (SDS-PAGE) and the resolved proteins were transferred to a polyvinylidene fluoride (PVDF) blotting membrane (#IPVH00010, Millipore Bioscience Research). To prevent non-specific protein binding, skim milk (#232100, BD DifcoTM) was added to 5% skim milk solution in Tris-buffered saline (TBS-T) containing 0.1% tween 20. Alternatively, BSA (#A2153-10G, SIGMA) was added to make a 2% bovine serum albumin solution in Tris-buffered saline (TBS-T) containing 0.1% tween 20 to make a blocking solution, and then reacted at room temperature for 1 h and 30 min. After the reaction, washing was performed thrice for 5 min with TBS-T, and the primary antibody was diluted with blocking solution and incubated at 4°C for 20 h. The primary antibody information and ratios used are as follows: 65 kDa, 1:5,000, rabbit monoclonal anti-yes associated protein 1 (YAP, #14074, Cell Signaling), 65–78 kDa, 1:5,000, mouse monoclonal anti-NF κ B p65 protein (#6956, Cell Signaling), 28–37 kDa, 1:10,000, BDNF (#ab108319, abcam) 43 kDa, 1:5,000, β -actin (#sc-47778, Santa Cruz Biotechnology). After washing thrice for 5 min with TBS-T, the secondary antibody was diluted with TBS-T and incubated at room temperature for 1 h. The secondary antibody information and ratios used were as follows: goat anti-rabbit IgG-H + I HRP-conjugated (#A120-101P, Bethyl antibodies) and goat anti-mouse IgG-H + I HRP-conjugated (#A90-116P, Bethyl antibodies). After the reaction, washing was performed thrice for 10 min each time with TBS-T. Finally, HRP was activated and visualized by chemiluminescence using an enhanced peroxidase detection kit (#EBP-1071; ELPIS-Biotech) for 1 min. Data were analyzed using ImageJ software and GraphPad Prism 9.

#### Lipopolysaccharide (LPS) treatment

After removing the culture medium of astrocytes cultured in PDL-cover slips, Ti, and GO-Ti in 6-well plates (SPL) or 24-well plates, the cells were washed twice with PBS. After which, 1 mg/mL lipopolysaccharide (LPS, #L4391, Sigma) was diluted 1/1,000 in astrocyte culture medium to a concentration of 1 μg/mL and incubated at 37°C and 5% CO_2_ for 4 h. The samples were then obtained, and each experiment was performed.

#### Data analysis and statistics

The data were analyzed using Microsoft Excel and Graphpad Prism V9.0. The normality of data was analyzed by the Shapiro-Wilk test. The statistical significance of the data was evaluated using unpaired *t*-test and one-way ANOVA test, and the data were expressed as the mean ± standard error of the mean (SEM). The significance levels are expressed as * (*p* < 0.05), ** (*p* < 0.01), *** (*p* < 0.001), and **** (*p* < 0.0001).

## Data Availability

The raw data supporting the conclusion of this article will be made available by the authors, without undue reservation.

## References

[B1] Adeel M. Bilal M. Rasheed T. Sharma A. Iqbal H. M. N. (2018). Graphene and graphene oxide: functionalization and nano-bio-catalytic system for enzyme immobilization and biotechnological perspective. Int. J. Biol. Macromol. 120, 1430–1440. 10.1016/j.ijbiomac.2018.09.144 30261251

[B2] Bramini M. Chiacchiaretta M. Armirotti A. Rocchi A. Kale D. D. Martin C. (2019). An increase in membrane cholesterol by graphene oxide disrupts calcium homeostasis in primary astrocytes. Small 15 (15), e1900147. 10.1002/smll.201900147 30891923

[B3] Cavalcanti-Adam E. A. Volberg T. Micoulet A. Kessler H. Geiger B. Spatz J. P. (2007). Cell spreading and focal adhesion dynamics are regulated by spacing of integrin ligands. Biophys. J. 92 (8), 2964–2974. 10.1529/biophysj.106.089730 17277192 PMC1831685

[B4] Chen L. Deng H. Cui H. Fang J. Zuo Z. Deng J. (2018). Inflammatory responses and inflammation-associated diseases in organs. Oncotarget 9 (6), 7204–7218. 10.18632/oncotarget.23208 29467962 PMC5805548

[B5] Chiacchiaretta M. Bramini M. Rocchi A. Armirotti A. Giordano E. Vazquez E. (2018). Graphene oxide upregulates the homeostatic functions of primary astrocytes and modulates astrocyte-to-neuron communication. Nano Lett. 18 (9), 5827–5838. 10.1021/acs.nanolett.8b02487 30088941

[B6] Choi S. S. Lee H. J. Lim I. Satoh J. Kim S. U. (2014). Human astrocytes: secretome profiles of cytokines and chemokines. PLoS One 9 (4), e92325. 10.1371/journal.pone.0092325 24691121 PMC3972155

[B7] Chung W. S. Allen N. J. Eroglu C. (2015). Astrocytes control synapse formation, function, and elimination. Cold Spring Harb. Perspect. Biol. 7 (9), a020370. 10.1101/cshperspect.a020370 25663667 PMC4527946

[B8] Cicchese J. M. Evans S. Hult C. Joslyn L. R. Wessler T. Millar J. A. (2018). Dynamic balance of pro- and anti-inflammatory signals controls disease and limits pathology. Immunol. Rev. 285 (1), 147–167. 10.1111/imr.12671 30129209 PMC6292442

[B9] Claramunt S. Varea A. Lopez-Diaz D. Velazquez M. M. Cornet A. Cirera A. (2015). The importance of interbands on the interpretation of the Raman spectrum of graphene oxide. J. Phys. Chem. C 119 (18), 10123–10129. 10.1021/acs.jpcc.5b01590

[B10] Colombo E. Farina C. (2016). Astrocytes: key regulators of neuroinflammation. Trends Immunol. 37 (9), 608–620. 10.1016/j.it.2016.06.006 27443914

[B11] Czeh M. Gressens P. Kaindl A. M. (2011). The yin and yang of microglia. Dev. Neurosci. 33 (3-4), 199–209. 10.1159/000328989 21757877

[B12] Delon I. Brown N. H. (2009). The integrin adhesion complex changes its composition and function during morphogenesis of an epithelium. J. Cell Sci. 122 (23), 4363–4374. 10.1242/jcs.055996 19903692 PMC2779134

[B13] Desai N. P. Hubbell J. A. (1991). Biological responses to polyethylene oxide modified polyethylene terephthalate surfaces. J. Biomed. Mater Res. 25 (7), 829–843. 10.1002/jbm.820250704 1833405

[B14] Dreyer D. R. Park S. Bielawski C. W. Ruoff R. S. (2010). The chemistry of graphene oxide. Chem. Soc. Rev. 39 (1), 228–240. 10.1039/b917103g 20023850

[B15] Dupont S. Morsut L. Aragona M. Enzo E. Giulitti S. Cordenonsi M. (2011). Role of YAP/TAZ in mechanotransduction. Nature 474 (7350), 179–183. 10.1038/nature10137 21654799

[B16] Fabbro A. Prato M. Ballerini L. (2013). Carbon nanotubes in neuroregeneration and repair. Adv. Drug Deliv. Rev. 65 (15), 2034–2044. 10.1016/j.addr.2013.07.002 23856411

[B17] Farina C. Aloisi F. Meinl E. (2007). Astrocytes are active players in cerebral innate immunity. Trends Immunol. 28 (3), 138–145. 10.1016/j.it.2007.01.005 17276138

[B18] Farina C. CaC. (2010). Astrocytes exert and control immune responses in the brain. Curr. Immunol. Rev. 6 (3), 150–159. 10.2174/157339510791823655

[B19] Fraczek-Szczypta A. Jantas D. Ciepiela F. Grzonka J. (2020). Graphene oxide-conductive polymer nanocomposite coatings obtained by the EPD method as substrates for neurite outgrowth. Diam. Relat. Mater, 102. 10.1016/j.diamond.2019.107663

[B20] Geim A. K. Novoselov K. S. (2007). The rise of graphene. Nat. Mater 6 (3), 183–191. 10.1038/nmat1849 17330084

[B21] Ghorbani F. Zamanian A. Aidun A. (2019). Bioinspired polydopamine coating-assisted electrospun polyurethane-graphene oxide nanofibers for bone tissue engineering application. J. Appl. Polym. Sci. 136 (24). 10.1002/app.47656

[B22] Giovannoni F. Quintana F. J. (2020). The role of astrocytes in CNS inflammation. Trends Immunol. 41 (9), 805–819. 10.1016/j.it.2020.07.007 32800705 PMC8284746

[B23] Guo S. B. Qu X. H. He X. B. Zhou T. Duan B. H. (2006). Powder injection molding of Ti-6Al-4V alloy. J. Mater Process Tech. 173 (3), 310–314. 10.1016/j.jmatprotec.2005.12.001

[B24] Han D. K. Park K. D. Ryu G. H. Kim U. Y. Min B. G. Kim Y. H. (1996). Plasma protein adsorption to sulfonated poly(ethylene oxide)-grafted polyurethane surface. J. Biomed. Mater Res. 30 (1), 23–30. 10.1002/(sici)1097-4636(199601)30:1<23:aid-jbm4>3.0.co;2-t 8788102

[B25] Hoyle C. Rivers-Auty J. Lemarchand E. Vranic S. Wang E. Buggio M. (2018). Small, thin graphene oxide is anti-inflammatory activating nuclear factor erythroid 2-related factor 2 via metabolic reprogramming. ACS Nano 12 (12), 11949–11962. 10.1021/acsnano.8b03642 30444603

[B26] Jensen C. J. Massie A. De Keyser J. (2013). Immune players in the CNS: the astrocyte. J. Neuroimmune Pharmacol. 8 (4), 824–839. 10.1007/s11481-013-9480-6 23821340

[B27] Jin J. Y. Zhao X. X. Fu H. F. Gao Y. (2020). The effects of YAP and its related mechanisms in central nervous system diseases. Front. Neurosci-Switz. 14, 595. 10.3389/fnins.2020.00595 PMC7333666 32676008

[B28] Jung H. S. Choi Y. J. Jeong J. Lee Y. Hwang B. Jang J. (2016). Nanoscale graphene coating on commercially pure titanium for accelerated bone regeneration. Rsc Adv. 6 (32), 26719–26724. 10.1039/c6ra03905g

[B29] Kate E. F. Tran N. L. Nguyen T. A. Nguyen T. T. Tran P. A. (2019). Surface modification of medical devices at nanoscale—Recent development and translational perspectives. Biomaterials Transl. Med., 163–189. 10.1016/b978-0-12-813477-1.00008-6

[B30] Lawrence T. (2009). The nuclear factor NF-kappa B pathway in inflammation. Csh Perspect. Biol. 1 (6), a001651. 10.1101/cshperspect.a001651 PMC2882124 20457564

[B31] Lee W. S. Kang J. H. Lee J. H. Kim Y. S. Kim J. J. Kim H. S. (2022). Improved gliotransmission by increasing intracellular Ca(2+) via TRPV1 on multi-walled carbon nanotube platforms. J. Nanobiotechnology 20 (1), 367. 10.1186/s12951-022-01551-1 35953847 PMC9367080

[B32] Lopez-Diaz D. Holgado M. L. Garcia-Fierro J. L. Velazquez M. M. (2017). Evolution of the Raman spectrum with the chemical composition of graphene oxide. J. Phys. Chem. C 121 (37), 20489–20497. 10.1021/acs.jpcc.7b06236

[B33] Luo X. Weaver C. L. Zhou D. D. Greenberg R. Cui X. T. (2011). Highly stable carbon nanotube doped poly(3,4-ethylenedioxythiophene) for chronic neural stimulation. Biomaterials 32 (24), 5551–5557. 10.1016/j.biomaterials.2011.04.051 21601278 PMC3109196

[B34] Marcin Behunova D. Gallios G. Girman V. Kolev H. Kanuchova M. Dolinska S. (2021). Electrophoretic deposition of graphene oxide on stainless steel substrate. Nanomater. (Basel) 11 (7), 1779. 10.3390/nano11071779 PMC8308149 34361165

[B35] Martino F. Perestrelo A. R. Vinarsky V. Pagliari S. Forte G. (2018). Cellular mechanotransduction: from tension to function. Front. Physiol. 9, 824. 10.3389/fphys.2018.00824 30026699 PMC6041413

[B36] Norden D. M. Trojanowski P. J. Villanueva E. Navarro E. Godbout J. P. (2016). Sequential activation of microglia and astrocyte cytokine expression precedes increased Iba-1 or GFAP immunoreactivity following systemic immune challenge. Glia 64 (2), 300–316. 10.1002/glia.22930 26470014 PMC4707977

[B37] Oh J. S. Jang J. H. Lee E. J. (2021). Electrophoretic deposition of a hybrid graphene oxide/biomolecule coating facilitating controllable drug loading and release. Metals-Basel 11 (6), 899. 10.3390/met11060899

[B38] Pan C. J. Pang L. Q. Gao F. Wang Y. N. Liu T. Ye W. (2016). Anticoagulation and endothelial cell behaviors of heparin-loaded graphene oxide coating on titanium surface. Mat. Sci. Eng. C-Mater. 63, 333–340. 10.1016/j.msec.2016.03.001 27040227

[B39] Parandeh S. Kharaziha M. Karimzadeh F. Hosseinabadi F. (2020). Triboelectric nanogenerators based on graphene oxide coated nanocomposite fibers for biomedical applications. Nanotechnology 31 (38), 385402. 10.1088/1361-6528/ab9972 32498060

[B40] Perez-Alvarez A. Araque A. (2013). Astrocyte-neuron interaction at tripartite synapses. Curr. Drug Targets 14 (11), 1220–1224. 10.2174/13894501113149990203 23621508

[B41] Perumbilavil S. Sankar P. Rose T. P. Philip R. (2015). White light Z-scan measurements of ultrafast optical nonlinearity in reduced graphene oxide nanosheets in the 400-700 nm region. Appl. Phys. Lett. 107 (5). 10.1063/1.4928124

[B42] Phatnani H. Maniatis T. (2015). Astrocytes in neurodegenerative disease: table 1. Cold Spring Harb. Perspect. Biol. 7 (6), a020628. 10.1101/cshperspect.a020628 25877220 PMC4448607

[B43] Place E. S. Evans N. D. Stevens M. M. (2009). Complexity in biomaterials for tissue engineering. Nat. Mater 8 (6), 457–470. 10.1038/nmat2441 19458646

[B44] Qin H. Gong T. Cho Y. Lee C. Kim T. (2014). A conductive copolymer of graphene oxide/poly(1-(3-aminopropyl)pyrrole) and the adsorption of metal ions. Polym. Chem-Uk 5 (15), 4466–4473. 10.1039/c4py00102h

[B45] Ruiz O. N. Fernando K. A. S. Wang B. J. Brown N. A. Luo P. G. McNamara N. D. (2011). Graphene oxide: A nonspecific enhancer of cellular growth. Acs Nano 5 (10), 8100–8107. 10.1021/nn202699t 21932790

[B46] Sidambe A. T. (2014). Biocompatibility of advanced manufactured titanium implants-A review. Materials 7 (12), 8168–8188. 10.3390/ma7128168 28788296 PMC5456424

[B47] Sidambe A. T. Figueroa I. A. Hamilton H. G. C. Todd I. (2012). Metal injection moulding of CP-Ti components for biomedical applications. J. Mater Process Tech. 212 (7), 1591–1597. 10.1016/j.jmatprotec.2012.03.001

[B48] Variola F. Vetrone F. Richert L. Jedrzejowski P. Yi J. H. Zalzal S. (2009). Improving biocompatibility of implantable metals by nanoscale modification of surfaces: an overview of strategies, fabrication methods, and challenges. Small 5 (9), 996–1006. 10.1002/smll.200801186 19360718

[B49] Wang J. Wang H. Mo X. Wang H. (2020). Reduced graphene oxide-encapsulated microfiber patterns enable controllable formation of neuronal-like networks. Adv. Mater 32 (40), e2004555. 10.1002/adma.202004555 32875631 PMC10865229

[B50] Wu S. Y. An S. S. Hulme J. (2015). Current applications of graphene oxide in nanomedicine. Int. J. Nanomedicine 10, 9–24. (Spec Iss). 10.2147/ijn.s88285 26345988 PMC4554423

[B51] Yao X. Yan Z. Wang X. Jiang H. Qian Y. Fan C. (2021). The influence of reduced graphene oxide on stem cells: A perspective in peripheral nerve regeneration. Regen. Biomater. 8 (4), rbab032. 10.1093/rb/rbab032 34188955 PMC8226110

[B52] Zhang B. Wang Y. Zhai G. (2016). Biomedical applications of the graphene-based materials. Mater Sci. Eng. C Mater Biol. Appl. 61, 953–964. 10.1016/j.msec.2015.12.073 26838925

[B53] Zuo J. Huang X. Z. Zhong X. X. Zhu B. S. Sun Q. Jin C. Y. (2013). A comparative study of the influence of three pure titanium plates with different micro- and nanotopographic surfaces on preosteoblast behaviors. J. Biomed. Mater Res. A 101 (11), 3278–3284. 10.1002/jbm.a.34612 23625827

