# Supplementary figures and images for: Regulation of astrocyte activity and immune response on graphene oxide-coated titanium by electrophoretic deposition
Source: Front Bioeng Biotechnol. 2023 Oct 3;11:1261255. doi: 10.3389/fbioe.2023.1261255 (PMC10579947; doi:10.3389/fbioe.2023.1261255)

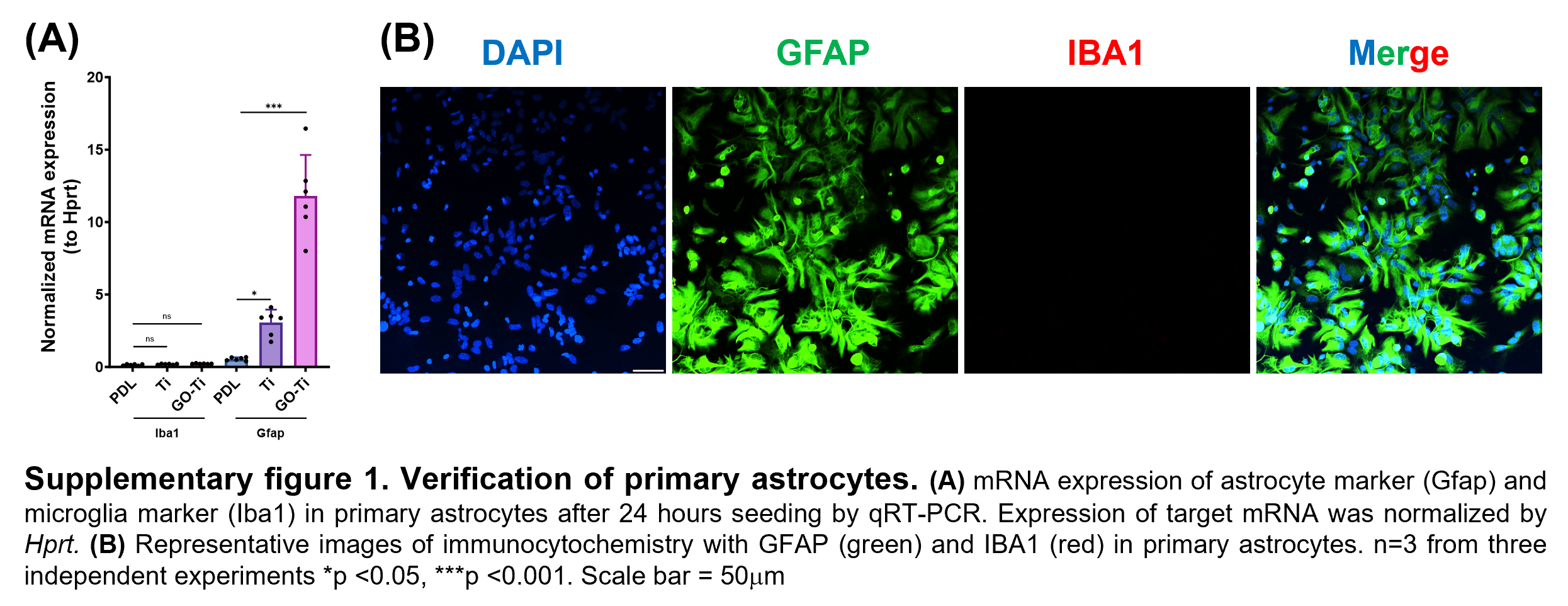

Supplement: Supplementary file 1 [file Image1.TIF]
